# Sleep quality and job satisfaction in Spanish nurses: the consequences of COVID-19

**DOI:** 10.15649/cuidarte.3452

**Published:** 2024-07-30

**Authors:** Alba Maestro-González, David Zuazua-Rico, Carmen Juan-García, Salvador Villalgordo-García, María Pilar Mosteiro-Díaz, María Plaza-Carmona

**Affiliations:** 1 Hospital Universitario Central de Asturias. Instituto de Investigación del Principado de Asturias. Oviedo. Spain. E-mail: albamaestrog@gmail.com Hospital Universitario Central de Asturias Instituto de Investigación del Principado de Asturias Oviedo Spain albamaestrog@gmail.com; 2 Faculty of Medicine and Health Sciences. University of Oviedo. Hospital Universitario Central de Asturias. Oviedo. Spain. E-mail: zuazuadavid@uniovi.es Universidad de Oviedo Faculty of Medicine and Health Sciences University of Oviedo Hospital Universitario Central de Asturias Oviedo Spain zuazuadavid@uniovi.es; 3 León University Hospital, León, Spain. E-mail: cjuangarcia@saludcastillayleon.es León University Hospital León Spain cjuangarcia@saludcastillayleon.es; 4 Hospital Universitario Central de Asturias. Oviedo. Spain. E-mail: salvillalgordo@gmail.com Hospital Universitario Central de Asturias Oviedo Spain salvillalgordo@gmail.com; 5 Faculty of Medicine and Health Sciences. University of Oviedo. Oviedo. Spain. E-mail: mmosteirod@uniovi.es Universidad de Oviedo Faculty of Medicine and Health Sciences University of Oviedo Oviedo Spain mmosteirod@uniovi.es; 6 León University Hospital, León, Spain. E-mail: miplazac@saludcastillayleon.es León University Hospital León Spain miplazac@saludcastillayleon.es

**Keywords:** COVID-19, Sleep, Sleep Hygiene, Job Satisfaction, Nursing, COVID-19, Sueño, Higiene del Sueño, Satisfacción en el Trabajo, Enfermería, COVID-19, Sono, Higiene do Sono, Satisfagáo no Emprego, Enfermagem

## Abstract

**Introduction:**

Sleep is fundamental to the quality of life and can affect individuals' well-being and mental health.

**Objective:**

This study analyzed sleep quality and job satisfaction among Spanish nurses following the COVID-19 pandemic.

**Materials and Methods:**

A cross-sectional study was conducted using the Pittsburgh Sleep Quality Index (PSQI), Font Roja Job Satisfaction Questionnaire, and sociodemographic and work- related variables. A “snowball” sampling method was employed through social media dissemination.

**Results:**

The mean PSQI score was 9.75 ± 4.36 points. The poorest sleep quality was identified in participants without dependents (p=0.031; p=3.329; 95% CI=0.035-6.354) and those with dependents other than children (p=0.022; p=4.121; 95% CI=0.575- 7.667). However, having a Ph.D degree (p=0.001; p=-3.406; 95% CI=-5.503- 1.309) or specialist degree (p=0.021; p=-1.639; 95% CI=-3.031- -0.247) was associated with better sleep quality. Higher job satisfaction was found among women (p=0.034; p=0.104; 95% CI=0.007-0.201) who did not work with COVID-19 patients (p=0.049; p=-0.085; 95% CI=-0.174- -0.003).

**Discussion:**

Improving working conditions, such as the nurse-to-patient ratio and distribution of work shifts, is crucial to enhancing nurses' sleep quality.

**Conclusions:**

Spanish nurses reported poor sleep quality. Not having dependents or having dependents other than children were risk factors for poor sleep quality. Job satisfaction was higher among women who did not work with COVID-19 patients. No relationship was found between job satisfaction and sleep quality.

## Resumen

**Introducción:**

El sueño es fundamental para la calidad de vida y puede afectar el bienestar y la salud mental de los individuos.

**Objetivo:**

Analizar la calidad del sueño y satisfacción laboral en enfermeras españolas tras la pandemia por COVID-19.

**Materiales y Métodos:**

Estudio transversal utilizando el Pittsburgh Sleep Quality Index (PSQI), el Cuestionario de Satisfacción Laboral Font Roja y variables sociodemográficas y laborales. Se empleó un muestreo en "bola de nieve" mediante divulgación en redes sociales.

**Resultados:**

La puntuación media del PSQI fue de 9.75 ± 4.36 puntos. La peor calidad del sueño se identificó en participantes sin personas a cargo (p=0,031;P=3,329;IC95%=0,035-6,354) y en aquellos con dependientes distintos de los hijos (p=0,022;P=4,121;IC95%= 0,575-7,667). Sin embargo, ser doctor (p=0,001;P=-3,406; IC95%=-5,503-1,309) o especialista (p=0,021;P=-1,639; IC95%=-3,031- -0,247) se asoció con una mayor calidad del sueño. La mejor satisfacción laboral se identificó en mujeres (p=0,034;P=0,104;IC95%=0,007-0,201), que no trabajaban con pacientes COVID (p=0,049;P=- 0,085;IC95%=-0,174- -0,003).

**Discusión:**

Es crucial mejorar las condiciones laborales, como la ratio enfermera-paciente y la distribución de los turnos de trabajo, para mejorar la calidad del sueño de las enfermeras.

**Conclusiones:**

Las enfermeras españolas presentan una mala calidad del sueño. No tener personas a cargo y tener dependientes distintos de los hijos fueron factores de riesgo de mala calidad del sueño. La satisfacción laboral fue mayor en mujeres que no trabajaban con pacientes COVID-19. No se encontró relación entre la satisfacción laboral y la calidad del sueño de los trabajadores.

## Resumo

**Introdujo:**

O sono é fundamental para a qualidade de vida e pode afetar o bem-estar e a saúde mental dos individuos.

**Objetivo:**

Este estudo analisou a qualidade do sono e a satisfago no trabalho entre enfermeiros espanhóis após a pandemia de COVID-19.

**Materiais e Métodos:**

Foi realizado um estudo transversal utilizando o Índice de Qualidade do Sono de Pittsburgh (PSQI), o Questionário de Satisfago no Trabalho Font Roja e variáveis sociodemográficas e relacionadas ao trabalho. Um método de amostragem “bola de neve”foi empregado por meio de divulgado em redes sociais.

**Resultados:**

A pontuagáo média do PSQI foi de 9.75 ± 4.36 pontos. A pior qualidade do sono foi identificada nos participantes sem dependentes (p=0,031; p=3,329; IC 95%=0,035- 6,354) e naqueles com outros dependentes que náo sejam filhos (p=0,022; P=4,121; IC 95%=0,575- 7,667). Entretanto, possuir doutoramento (p=0,001; P=-3,406; IC 95%=-5,503-1,309) ou especialista (p=0,021; P=-1,639; IC 95%=-3,031- -0,247) esteve associado a melhor qualidade do sono. Maior satisfagáo no trabalho foi encontrada entre mulheres (p=0,034; p=0,104; IC 95%=0,007- 0,201) que náo trabalhavam com pacientes com COVID-19 (p=0,049; P=-0,085; IC 95%=-0,174- -0,003).

**Discussao:**

Melhorar as condigóes de trabalho, tais como o rácio enfermeiro/paciente e a distribuido dos turnos de trabalho, é crucial para melhorar a qualidade do sono dos enfermeiros.

**Condusdes:**

Os enfermeiros espanhóis relataram má qualidade do sono. Náo ter dependentes ou ter outros dependentes além dos filhos foram fatores de risco para má qualidade do sono. A satisfagáo no trabalho foi maior entre as mulheres que náo trabalharam com pacientes com COVID-19. Nenhuma relagáo foi encontrada entre satisfagáo no trabalho e qualidade do sono.

## Introduction

Historically, healthcare professionals have prioritized patient care at the expense of their own rest. They often endure long shifts with little sleep, work rotating shifts without fully understanding the implications, and expose themselves to critical situations arising from illness and emergencies^1^^, ^^4^.

Partial sleep loss has been linked to increased physiological stress responses, suggesting that shift workers are more susceptible to developing burnout syndrome^5^^, ^^6^. Several studies have indicated that the nursing staff are among the groups with the highest rates of burnout, work-related stress, and workload, likely because they spend the most time with patients and are responsible for providing care around the clock^7^^, ^^8^.

In terms of rest, all aspects of sleep were significantly affected by rotating shifts, including total sleep time, perceived sleep quality (well-being and satisfaction with sleep), restorative sleep (feeling physically refreshed upon waking), and nighttime awakenings (an index of sleep fragmentation). These findings align with studies describing that rotating-shift workers experience poorer sleep quality^7^^, ^^9^ and more sleep disturbances, such as insomnia, snoring, and excessive daytime sleepiness, than workers on regular shifts^10^. Sleep quality is critical not only for health but also for quality of life, which is another factor that rotating shift workers often report as compromised^11^.

Tolerance of shift work is the ability to adapt to rotating shifts without adverse consequences. The effects of shift work can be biological (metabolic disruptions^12^, hormonal imbalances, sleep cycle disturbances, cardiovascular disorders^7^, gastrointestinal issues, certain types of cancer^13^^, ^^14^, impaired mental agility, concentration, and reaction time), occupational (fluctuations in performance, errors, traffic accidents, and increased absenteeism)^15^, and social (difficulty balancing family responsibilities and limited time for social activities). Tolerance or adaptation to shift work depends on various personal characteristics, coping strategies, family and social conditions, work conditions, and, in particular, the organization of work hours. The outcomes of these interactions depend on the specific burden of each factor and its temporal patterns in the worker's life. Consequently, adaptation or tolerance to shift work is often assessed through symptoms, which indicate how many aspects of worker health and quality of life are affected^16^.

Moreover, chronic work stress during the COVID-19 pandemic exacerbated fatigue^17^. Healthcare professionals experience negative attitudes and feelings toward colleagues and their professional roles, leading to emotional exhaustion and burnout syndrome^7^^, ^^18^. The high workload, increased demand, and limited social support during the pandemic may have contributed to lower job satisfaction among nurses due to constant stress, shift changes, variations in work assignments, insufficient safety protocols, and shortages of resources, both human and material. These factors can cause biochemical and psychological alterations^19^. This study aimed to analyze the sleep quality and job satisfaction of Spanish nurses after the COVID-19 pandemic.

## Materials and Methods

### A Design

This descriptive cross-sectional study used data collected between March and April 2022.

### Participants

The study was conducted among all Spanish nurses who used social networks and agreed to participate. No sample size was estimated because the study aimed to include the entire population. Inclusion criteria required participants to be in active nursing practice throughout the data collection period, regardless of the department in which they worked or their age. Exclusion criteria included participants diagnosed with a sleep disorder, those on sick leave due to a stressful event according to the DSM-5 diagnostic criteria^20^, or those undergoing treatment for elevated stress levels. A snowball sampling technique was employed, encouraging participants to share and distribute the questionnaire through their social media networks and professional connections, which allowed for broader distribution and a more diverse participant base. Invitations to participate in the study were sent via Twitter, Instagram, and WhatsApp. Following the initial dissemination, the snowball sampling approach was used to further encourage participants to share the questionnaire on their own social media profiles.

### Data collection

Nurses who decided to participate in the project completed an online self-administered questionnaire. For this purpose, we designed a data collection sheet using Google Forms and distributed it on social media. The form included an introductory paragraph informing the participants about the study's objective and explaining that completing the questionnaire implied their consent to participate in the research. Anonymity was ensured by not collecting personal data. This instrument included sociodemographic variables (age, sex, relationship status, and dependents), work-related variables (education level, professional experience, shift type, department, and whether they worked with COVID-19 patients), the Spanish version of the Pittsburgh Sleep Quality Index (PSQI)^21^, and the Font Roja Job Satisfaction Questionnaire^22^. The database was stored in Zenodo^23^.

### Ethical considerations

This study was designed in accordance with the principles outlined in the Declaration of Helsinki^24^, the Belmont Report^25^, the CIOMS Guidelines^26^ and the provisions of Spain's Organic Law 3/2018, dated December 5, concerning the Protection of Personal Data and the Guarantee of Digital Rights. This study was approved by the León and Bierzo Drug Research Ethics Committee (No. 2021/2193). Furthermore, all study participants provided informed consent and the study procedures complied with the provisions of the Organic Law on Personal Data Protection.

### Data analysis

A descriptive analysis of each variable was performed to describe the demographic characteristics. Differences between the two groups were assessed using Student's t-test (with Welch's correction for different variances) or Wilcoxon's test for independent samples since the assumptions of normality (Shapiro-Wilk test), and homoscedasticity (Bartlett test and Ansari-Bradley test) could not be verified. Quantitative variables among the three categories were compared using the Kruskal-Wallis test and Dunn's post hoc test. Spearman's correlation coefficient and the corresponding hypothesis test were used to evaluate the linear relationship between the continuous variables. Finally, a linear model was constructed using variables with a p-value <0.10 in the bivariate analysis. Statistical significance was set at p < 0.05. Statistical analysis was performed using R software (R Development Core Team), version 4.4.0.

### Validity and reliability/Rigor

The PSQI is one of the most widely used instruments for assessing sleep quality due to its ease of self-administration^27^. It is a 24-item questionnaire that evaluates seven components (regular sleep efficiency, sleep latency [the time taken to transition from wakefulness to sleep], total sleep duration, sleep quality, use of sleep medication, daytime dysfunction, and sleep disturbances) from which a final score is derived. The PSQI uses a Likert-type scale ranging from 0 to 3. For correction, a sleep profile was obtained for each component, ranging from 0 to 3, and an overall score ranging from 0 to 21. Higher scores indicated poorer sleep quality, whereas scores of 5 or below indicated good sleep.

The study also used The Font-Roja Job Satisfaction Questionnaire^22^. Similar to the PSQI, it employs a Likert-type scale to evaluate its 24 items divided into nine factors that assess different aspects of job satisfaction: general job satisfaction, job-related stress, job competition, job pressure, job promotion opportunities, interpersonal relationships with superiors, interpersonal relationships with peers, extrinsic status characteristics, and job monotony. The overall mean satisfaction score was obtained by summing up the 24 items and calculating the arithmetic mean. The answers to the set ofquestions for each factor followed a Likert scale ranging from 1 to 5 (strongly disagree, disagree, neither agree nor disagree, agree, and strongly agree). The score obtained for each factor is equal to the sum of the scores of its sections, divided by the number of sections, each section with values ranging from 1 to 5. This questionnaire does not have a reference scoring criterion, so the interpretation of the results relies on data from relevant literature.

## Results

In total, 523 questionnaires were collected, constituting a representative sample of the national population. Regarding participant characteristics, 85.47% were women, with a mean age of 36.9±10.6 years. The majority held a bachelor's degree (56.65%), worked in hospitals (68.84%), and had fixed morning or afternoon shifts (48.83%). The average professional experience was 14.1±10.6 years; 77.53% worked with COVID-19 patients, 72.47% had a partner, and 63.72% did not have dependents (Table 1).

**Table 1:** Participants’ demographics and work information

Characteristics	% (n) (523)
	
Sex	
Male	14.53(76)
Female	85.47(447)
Age	
Mean ± SD	36.87 ± 10.56
Min/Max	22/65
Professional experience. Years	
Mean ± SD	14.14 ± 10.57
Min/Max	0.6/44
Civil Status	
With a partner	72.47 (379)
Single	27.53 (144)
Dependents	
Both	2.15 (11)
No	63.72 (333)
Yes, children	30.84 (161)
Yes, other people	3.41 (18)
Educational attainment	
Graduate	56.65 (296)
PhD	3.82 (20)
Nurse Practitioner	11.51 (60)
Master's Degree	28.12 (147)
Work Center	
Primary Care Center	21.17 (110)
Private center	5.03 (26)
Emergency	1.87 (10)
Hospital	68.84 (359)
Other Centers	3.36 (17)
Service	
Primary Care	23.31 (110)
Critical Care Unit	14.83 (70)
Outpatient Services	12.92 (61)
Inpatient unit	33.69 (159)
Emergency	15.25 (72)
Rotating work shifts	
Fixed shift	48.83 (254)
On-call duty	4.44 (23)
Rotating work Shift	46.80 (244)
Working with COVID-19 patients	77.53 (338)

The mean score for the Pittsburgh Sleep Quality Index (PSQI) among the nursing staff was 9.75±4.36, indicating poor sleep quality, as scores of 5 or above denote poor sleep quality. When evaluating the different components of the PSQI, the component with the worst score was sleep latency, with a mean of 2.84 ± 1.81. Other components, in ascending order of severity, were subjective sleep quality (1.73 ±1.25), daytime dysfunction (1.64 ±0.85), sleep disturbances (1.43 ±0.58), habitual sleep efficiency (1.07 ±1.07), use of sleep medication (0.62 ±1.02), and sleep duration (0.43 ±0.55). In terms of actual sleep time, the nursing staff reported an average of 5.97 hours of rest per night (±1.10; range 3-10 hours) (see Table 2).

**Table 2:** Comparison of perceptions of COVID-19 regarding mode of transmission, incubation period, symptoms, risk factors, prevention initiatives, and challenges by sex and age group

N		Mean	Min	Max
PSQI				
Item 1: Subjective sleep quality	523	1.73 ±1.25	0	3
Item 2: Sleep latency	523	2.84 ±1.81	0	6
Item 3: Sleep duration	517	0.43 ±0.55	0	2
Item 4: Habitual sleep efficiency	513	1.07 ±1.07	0	3
Item 5: Sleep disturbance	523	1.43 ±0.58	0	3
Item 6: Use of sleep medication and daytime dysfunction	523	0.62 ±1.02	0	3
Item 7: Dysfunction during the day	523	1.64 ±0.85	0	3
Global PSQI	523	9.75 ±4.36	1	20
Font Roja				
FACTOR 1. Job satisfaction	523	3.76 ±0.63	1.75	5
FACTOR 2. Work-related stress	523	3.01 ±0.83	1	5
FACTOR 3. Professional competence	523	2.56 ±0.80	1	5
FACTOR 4. Pressure at work	523	3.23 ±1.09	1	5
FACTOR 5: Career advancement	523	3.42 ±0.74	1	5
FACTOR 6. Interpersonal relationship with superiors	523	3.46 ±0.85	1	5
FACTOR 7. Interpersonal relationship with peers	523	2.32 ±1.22	1	5
FACTOR 8. Extrinsic status characteristics	523	2.94 ±0.76	1	5
FACTOR 9. Work monotony	523	2.48 ±0.89	1	5
FACTOR 10. Physical work environment satisfaction	523	2.06 ±0.90	1	5
Global satisfaction	523	2.98 ±0.35	1.77	4.04

A statistical association was found between the overall PSQI scores and several variables. Participants with poor sleep quality included those without dependents (p = 0.016; Dunn's test: p = 0.004), those with a master's degree (p < 0.001; Dunn's test: p = 0.001), those working in hospital care (p = 0.042; Dunn's test: p = 0.049), those caring for COVID-19 patients (p = 0.013), and those on rotating shifts (p = 0.011; Dunn's test: p = 0.008) (Table 3).

**Table 3:** Bivariate analysis of sleep quality and job satisfaction

	Mean ± SD	P (rho)	Mean ± SD	P (rho)
Age		0.423 (rho -0.035)		0.984 (rho 0,001)
Professional experience		0.279 (rho -0.031)		0.690 (rho 0.011)
Sex		0.329**		0.013*
Male	9.22 ± 4.20		2.88 ± 0.38	
Female	9.84 ± 4.37		3.00 ± 0.35	
Civil Status		0.429**		0.005**
With a partner	9.65± 4.38		2,98 ± 0.35	
Single	10.01± 4.27		2,94 ± 0.36	
Dependents		0.016#		0.578#
Both	7.36± 4.03		2.90 ± 0.50	
No	10.07± 4.46		2.97 ± 0.33	
Yes, children	9.11 ± 4.03		3.01 ± 0.38	
Yes, other people	11.11± 4.40		2.95 ± 0.46	
Educational attainment		<0.001#		0.633#
Graduate	10.04± 4.21		2.97 ± 0.37	
PhD	6.90± 4.19		3.04 ± 0.31	
Nurse Practitioner	7.80± 3.95		3.02 ± 0.29	
Master's Degree	10.35± 4.49		2.96 ± 0.35	
Work Centre		0.042#		0.163#
Primary Care Center	8.84± 4.07		2.99 ± 0.36	
Private Center	9.15± 3.51		2.84 ± 0.40	
Emergency	10.20± 5.33		2.83 ± 0.48	
Hospital	10.13± 4.42		2.99 ± 0.35	
Other Centers	8.35± 4.66		2.94 ± 0.41	
Service		0.124#		0.018#
Primary Care	8.83± 4.08		2.99 ± 0.36	
Critical Care Unit	9.87± 4.08		2.89 ± 0.35	
Outpatient Services	9.73± 4.37		2.95 ± 0.40	
Inpatient unit	10.17± 4.62		3.01 ± 0.32	
Emergency	10.26± 4.43		3.08 ± 0.34	
Rotating work shifts		0.011#		0.270#
Fixed shift	9.20± 4.24		3.01 ± 0.38	
On-call duty	9.61± 4.72		2.94 ± 0.37	
Rotating work Shift	10.34± 4.34		2.95 ± 0.33	
Working with COVID-19 patients		0.013**		0.038**
No	8.94± 4.33)		3.04 ± 0.37	
Yes	10.11± 4.35		2.96 ± 0.34	

PSQI Total Score Job Satisfaction

* Student's t-test; ** Wilcoxon's test; # Kruskal-Wallis test

A linear model was constructed to predict the sleep quality. The only variables that showed a statistically significant association were the presence of dependents and the highest level of education. The model explained 5.5% of the variability in sleep quality and was statistically significant (adjusted R-squared: 0.055; p = 0.003). Participants without or with dependents other than children had higher PSQI scores, indicating poor sleep quality. Conversely, obtaining a PhD or specialist degree was associated with better sleep quality (Table 4).

**Table 4:** Linear models

Coefficients	Sleep Quality Linear Model		Job Satisfaction Linear Model	
ß (95% CI)	p-value	ß (95% CI)	p-value
Sex				
Male	0	0	0	0
Female	0,705 (-0.44 - 1.858)	0,230	0.104 (0.007 - 0.201)	0.034
Age	0,040 (-0.005 - 0.087)	0,086	-0.001 (-0.004 - 0.002)	0.564
Civil Status				
With a partner			0	0
Single			0.014 (-0.064 -0.092)	0.721
Dependents				
Both	0	0		
No	3,329 (0.305 - 6.354)	0,031		
Yes, children	2,352 (-0.594 -5.300)	0,117		
Yes, other people	4,121 (0.575 - 7.667)	0,022		
Educational attainment				
Graduate	0	0		
PhD	-3,406 (-5.503 - -1.309)	0,001		
Nurse Practitioner	-1,639 (-3.031 - -0.247)	0,021		
Master's Degree	0,192 (-0.760 - 1.145)	0,692		
Work Centre				
Primary Care Center	0	0		
Private center	-0,049 (-2.118 - 2.019)	0,962		
Emergency	0,594 (-2.753 - 3.942)	0,727		
Hospital	0,690 (-0.486 -1.868)	0,249		
Other Centers	-0,052 (-2.443 -2.33)	0,965		
Service				
Primary Care			0	0
Critical Care Unit			-0.103 (-0.215 -0.009)	0.072
Outpatient Services			-0.040 (-0.164 -0.083)	0.520
Inpatient unit			0.010 (-0.084 - 0.104)	0.832
Emergency			0.073 (-0.039 -0.186)	0.200
Rotating work shifts				
Fixed shift	0	0		
On-call duty	-0,050 (-2.361 - 2.259)	0,965		
Rotating work Shift	0,789 (-0.178 - 1.756)	0,109		
Working with COVID-19 patients				
No	0	0	0	0
Yes	0,865 (-0.169 - 1.900)	0,101	-0.085 (-0.174 -0.003)	0.049
(Intercept)	3,491 (-0,512 - 7,495)	0,087	3,007 (2.818 - 3.197)	<0,001

The overall job satisfaction was 2.98 ± 0.35 points. The component with the lowest score was satisfaction with the physical work environment (2.06 ±0.90), followed by interpersonal relationships with colleagues (2.32 ±1.22), work monotony (2.48 ±0.89), professional competence (2.56 ±0.80), extrinsic status characteristics (2.94 ±0.76), job stress (3.01 ±0.83), job pressure (3.23 ±1.09), career advancement (3.42 ±0.63), interpersonal relationships with superiors (3.46 ±0.85), and job satisfaction (3.76 ±0.63) (Table 2).

A statistical association was found between the overall Font Roja Questionnaire scores and several variables. The lowest satisfaction levels were identified among men (p = 0.011), single workers (p = 0.005), those working in critical care (p = 0.018; Dunn's test: p = 0.047), and those caring for COVID-19 patients (p = 0.038) (Table 3).

A linear model was constructed to predict job satisfaction. The only statistically significant variables were sex and working with COVID-19 patients. The model explained 2.7% of the variability in job satisfaction and was statistically significant (Adjusted R-squared: 0.027; p = 0.016). Job satisfaction scores were higher among women who did not work with COVID-19 patients (Table 4). No significant relationship was found between overall job satisfaction and sleep quality (p = 0.359).

## Discussion

The COVID-19 pandemic has significantly affected healthcare, causing stress and anxiety among healthcare professionals^28^^, ^^30^. This situation has resulted in frequent disruptions in sleep patterns and has led to a poor perception of sleep quality among nurses^31^^, ^^32^. In this study, most participants exhibited poor sleep quality, with a mean Pittsburgh Sleep Quality Index (PSQI) score of 9.75.

Our findings are consistent with those of a study conducted in the United States^33^, where the mean PSQI score was 9.27, and poor subjective sleep quality was linked to low job satisfaction. Similarly, a study conducted among Chinese nurses^34^ showed that sleep quality is associated with psychological distress and job burnout (mean PSQI score = 9.10). Another study by Lyu et al.^35^, reported lower mean PSQI scores, but still indicated poor sleep quality (mean PSQI score of 7.00). This difference could be attributed to the lower exposure of COVID-19 patients, as the setting was psychiatric. Additionally, in an Italian study involving physicians and nurses^36^, researchers highlighted the negative impact of the pandemic on psychological and sleep-related aspects, especially among frontline nurses.

In Spain, the general population had a lower mean PSQI score (8.45)^37^ at the beginning of the pandemic than the score obtained in our study after two years, which could be due to nurse attrition over time. Similar results were found in an emergency department during the COVID-19 pandemic, with a comparable overall PSQI score of 8.27^38^. The poor perception of sleep quality among Spanish nurses is consistent with Moreno-Casbas’ findings^39^, which also noted poor sleep quality perception, with worse results for nurses working fixed night shifts (mean PSQI score = 7.93).

When assessing sleep quality across different services, special services yielded alarming values similar to those found in our study. Suleiman et al.^40^ reported a mean PSQI score of 8.76 in a sample of 200 emergency nurses. In the same context, it was noted that stress levels in Spain increased during the COVID-19 pandemic, with higher levels among those with poor sleep quality and increased sleepiness, both in the pre-pandemic phase and afterward^41^.

The mean age of our sample was 36 years, with a mean professional experience of 14 years. Factors potentially linked to physical and psychological strain resulting from shift work can lead to sleep disturbances. Studies such as those by Senol et al.^42^, in which the study sample was in its early years of work, had a lower average PSQI score (4.14), suggesting that the impact of shift work on sleep patterns might not yet have become apparent^7^. When examining the components of the PSQI, sleep latency had the lowest score (2.84), which aligns with the findings of Maestro-Gonzalez et al.^37^ during the lockdown period, in which sleep latency was also the lowest-rated component.

Despite these results, Kang et al.^43^ highlighted the need for assessment instruments tailored to shift work characteristics. The PSQI includes items such as average time to fall asleep and sleep duration at night, which may not be suitable for assessing sleep quality in shift workers. There is also an association between different circadian rhythm patterns and the risk of developing burnout, influenced by factors such as gender, work shift, service type, family environment, and education level, as noted in studies by Bagheri et al.^44^ and Giorgi et al.^45^.

In our study, we found significant differences in poor sleep quality among nurses with dependents and those with lower education levels. These results are similar to those obtained in Turkey, where higher education was associated with better sleep quality^42^. This contrasts with studies by Suleiman et al.^40^, McDowall et al.^9^ and Maestro-Gonzalez et al.^37^, in which sociodemographic variables did not significantly impact sleep quality. The reason for this discrepancy is unclear, indicating the need for qualitative research to explore the underlying causes.

Similarly, our study observed differences in occupational stress between sexes, with men experiencing higher stress levels, which differs from other studies^46^. When examining job satisfaction and stress, it was observed that stress due to workload can lead to difficulties in falling asleep and early or frequent awakenings, which can negatively affect sleep quality^47^. Continuous awakening was one of the most common symptoms reported in this study.

Our findings are consistent with those of Rahnavard^48^, who observed that professionals in special services, such as emergency departments, experienced lower job satisfaction. The worst satisfaction was identified among men who worked with COVID-19 patients, which aligns with Rouxel et al.^49^, who reported that high job demands were associated with increased emotional exhaustion and staff depersonalization. Burnout was more prevalent among professionals in intensive care units, possibly due to high physical and psychological pressures, consistent with other studies conducted during the COVID-19 pandemic^50^^, ^^51^. However, another study in Portugal conducted during the third wave of COVID-19 showed higher job satisfaction among nurses working in services for COVID-19 patients^52^.

Given these factors, our study emphasizes the need to improve working conditions, such as nurse-to- patient ratios and shift distribution, to enhance sleep quality among nurses and potentially reduce their stress and burnout.

Limitations of this study stem primarily from its cross-sectional design. It was not possible to analyze the evolution of the participants’ sleep quality and job satisfaction over time. Additionally, although our participant selection strategy yielded a representative sample of Spanish nurses, we may have excluded those without Internet access. Future studies should consider cohort studies to explore the long-term evolution of these aspects. Additionally, it would be interesting to identify other variables that influence these factors because their explanatory power is small.

## Conclusion

Spanish nurses experienced poor sleep quality following the COVID-19 pandemic, with sleep latency being the lowest-rated aspect. Risk factors for poor sleep quality included not having dependents or having dependents other than children while obtaining a doctorate or specialist degree served as a protective factor.

With regard to job satisfaction, the component with the lowest score was satisfaction with the physical work environment, followed by interpersonal relationships with colleagues. Factors that increased job satisfaction included being a woman and not working with COVID-19 patients. However, job satisfaction has decreased among men working in critical care. No significant relationship was observed between job satisfaction and sleep quality.

It is crucial to emphasize the importance of nurses' sleep quality to ensure that patients receive safe, high-quality care and that nurses experience greater satisfaction with their job performance and working conditions.
